# To the Editors of the Medical and Physical Journal

**Published:** 1801-10

**Authors:** 


### *To the Editors of the Medical and Phyfical Journal.*

GENTLEMEN,

IN a late Number of your Mifcellany, Dr. Crichton has related three cafes of jaundice produced by a fcirrhus of the pancreas, a caufe which he conceives to have been hitherto undefcribed by authors. I truft that he will not fufpect me of any intention to depreciate his valuable and interefting communication, if I point out to his notice the following extracts from a work of no inconfiderable merit or celebrity, the Hiftoria Anatomico-Medica of Lieutaud.

"Vir *quinquagenarius* dolore corripitur in abdomine fupra umbilicum; poft varias remiffiones, per duos menfes, cutis *colore icteritis* defœdatur. Dolores intereà fiunt acerbiores, et quarto ab *ictero* die tumet hypochondrium dextrum. Dein ferociora urgent tormina; mifellufque æger clamans et ejulans in convulfiones lethales incidit.

"Inter fectionem cadaveris reperitur *ductus biliferus mirum in modum a bile stagnante dilatatus*; adeo ut venæ portæ amplitudinem fuperaret. Veficula fellis ab ftatu naturali minime recedebat, fi excipias cervicem contractam, quæ appellenti bili aditum præcludebat. *Pars dextra pancreatis*, protuberans et premens ductûs cyftici oftium, *dura et scirrhosa* deprehenditur; finiftra verò putrida. Duodenum hujufce mali confors, callofum deprehenditur." Obf. 1012.

"Vir *sexagenarius*, vino fupra modum deditus, et ad tabem vergens, fubito in *Icterum* incidit. Tum vires deficiunt, arcetur fomnus; accedunt naufeæ cum ventris doloribus. Dein manus tremore debilitantur, fubfequente totali impotentiâ, quæ artus inferiores etiam invadit. Tandem exolutis viribus occubuit.

"Luftratis interaneis, deprehendebatur *Pancreas* tumidum, univerfali et obfirmato *scirrho induratum*. Cætera vifcera, non fecus ac cutis, bile infecta videbantur." Obf 1018.

Although there are fome circumftances in which the above cafes differ from thofe related by Dr. Crichton, particularly the abfence of diarrhœa, there are yet many points of ftriking coincidence. I fhould not however have troubled you on the fubject, but for an extraordinary letter in your laft Number, the writer of which (if we are to judge from his mode of expreffion) feems defirous of taking fome credit to himfelf, for having in his Thefis mentioned fcirrhous pancreas as a caufe of jaundice. Whatever merit this production may have in other refpects, it certainly has no claim to originality on that fcore; the circumftance has been mentioned in moft of the Edinburgh

Inaugural Diſſertations "de Ictero" for many years paſt; indeed, if it has ever been omitted, the authors muſt have been guilty of very culpable negligence, as Dr. Monro has long been accuſtomed annually to demonſtrate the preparation which your Correſpondent inſinuates to have been exhibited in "corroboration" of *his* ſtatement. I am, &c.

*Sept.* 12, 1801. M.

